# Response: Commentary: Health and safety practices and policies concerning human exposure to RF/microwave radiation

**DOI:** 10.3389/fpubh.2026.1815988

**Published:** 2026-03-25

**Authors:** James C. Lin

**Affiliations:** Department of Electrical and Computer Engineering, University of Illinois Chicago, Chicago, IL, United States

**Keywords:** guidelines and standards, harmonization of RF exposure standards, microwave auditory effect, pressure waves in brain, RF and microwave radiation, tissue temperature rises, traumatic brain injury, WHO systematic reviews

I thank Bailey, Foster and Chou for their letter (1) and interest in my recent paper (2). It is understood that they hold views that are different from mine on subject matters. And we may engage different styles for narratives and apply dissimilar approaches to reaching conclusions. I agree with the letter writers that the subject matters are complex, and the issues cannot be adequately addressed in brief letters or commentaries. Nevertheless, the following are my responses to their letter.

They claim that I misrepresented the ICES C95.1-2019 (3) and ICNIRP-2020 (4) promulgated RF limits for safe human exposure. Note that I do not pretend to represent either ICES C95.1 or ICNIRP. Instead, Table 1 briefly summarized the current ICES and ICNIRP recommended guidelines or standards for human exposure to RF radiation. My paper assessed and commented on some of their salient features. Specifically, it mentioned that “for frequencies between 6 GHz to 300 GHz including 5G cellular mobile communication, the exposure limits allow tissue temperature rises in the human head, limbs and torso to *as high as* 5°C.” Also, the letter writers acknowledge that the limits “are for all body surface tissues,” and pointed out “not separated as local head-torso and local limbs as in Table 1.” It should be clear that “all body surface tissues” would include the head, torso and limbs tissues since skins are constituents of the head, torso and limbs. Nonetheless, for completeness, interested readers are encouraged to consult the ICES and ICNIRP exposure guidelines or standards documents.

I regret not catching errors in power density unit designations in Table 1 during page proof. I thank the letter writers for spotting them. They should read as W/m^2^. Likewise, for the error on the power density units associated with the Dagro cite (5), the correct exposure unit is MW/m^2^, not W/m^2^.

As for the microwave auditory effect resulting from microwave pulse induced acoustic pressure waves inside the brain, it is critical to understand that the microwave auditory effects can be adverse and occur below 1°C [For a 10-μs microwave pulse, the calculated temperature is about 10^−6*o*^C at the threshold of human perception (6, 7)]. They claim “RF-induced sounds are similar to other common sounds.” This is simply not true. It suggests that the letter writers do not know much about the effect or have very poor understanding of it. The effect is not a common sound! The perceived sound does not travel from the outer ear nor through the ear canal (6, 7). The significant points in this case are: (1) microwave pulse produces substantial acoustic pressure waves within the brain that have implications for neuropathological consequences including the Havana syndrome (6–9) and startle responses (6, 10–12) and (2) the effect falls within the permissible “safe” limits of currently promulgated exposure standards and protection guidelines.

Furthermore, the Dagro et al. study (5) showed that to generate tissue injuring levels of microwave induced acoustic pressure waves inside the human brain, the microwave pulse induced temperature rises would be substantially below the putative “safe” threshold (1°C). Therefore, the exposure would be allowable according to currently promulgated ICES C95.1 and ICNIRP RF safety limits. It is noteworthy that for sufficiently high incident power densities, a single microsecond-pulse at significantly below the current standards could potentially result in biologically significant pressures; the small, induced temperature rise (< 0.0005°C) may create injurious pressure waves for traumatic brain injury in humans (5, 8).

Global harmonization of RF exposure standards and guidelines is a reasonable goal. The process and result should be advancements beyond the status quo for better precision and lower uncertainty. Sadly, the harmonization process actually decreased precision and increased uncertainty. Also, it failed to recognize contemporary advances in RF and microwave safety protection science (2, 13).

As for grounded on a strong RF heating conviction, indeed, the letter quoted ICNIRP by saying: “The lowest exposure levels that can cause adverse health effects are due to thermal mechanisms, and so restrictions have been set based on the thermal effects, as these will protect against any other effects that could occur at higher exposure levels.”

It is unfortunate that statements such as “ICNIRP review did not identify reliable evidence for effects of RF exposure that would presently warrant a change in its guidelines” (ICNIRP-2025) (14) and “none reported consistent, confirmed health effects at levels below IEEE and ICNIRP exposure limits” (1) have become cliches, just like the phrase, “more systematic and in depth research is needed.”

A total of 12 SR's was published as a special series in the journal *Environment International* between 2023 and 2025. The protocols for the WHO-commissioned systematic reviews were published in 2021 and 2022. An introduction to the special series appeared in September 2025 following publication of the last of all 12 SRs. The topics chosen for the 12 SR's include cancer, birth outcomes, male fertility, oxidative stress, observable and self-reported symptoms, cognitive impairment, and electromagnetic hypersensitivity-related responses.

The letter complained that the “critique of World Health Organization (WHO) sponsored systematic reviews (SRs) relies on selected criticisms without evaluating their objectivity and substance. Consequently, his commentary lacks an adequate basis for its conclusions.”

In addition to the latest WHO SRs on effects of RF exposure on cancer in experimental animals (15), my paper (1) reviewed, assessed and commented on the first 4 SRs as examples, alongside syntheses of observations made by many other scientists. I stand by my evaluation, comments and conclusions. For interested readers, a recent paper (16) critically evaluated all 12 WHO-SRs on health effects of RF and microwave radiation.

## References

[1] Bailey WH Foster KR Chou CK. Commentary: Health and safety practices and policies concerning human exposure to RF/microwave radiation. Front Public Health. (2026) 14:1752150. doi: 10.3389/fpubh.2026.1752150 41799448 PMC12961997

[2] Lin JC. Health and safety practices and policies concerning human exposure to RF/microwave radiation. Front Public Health. (2025) 13:1619781. doi: 10.3389/fpubh.2025.1619781 40761932 PMC12318757

[3] IEEE Std. C95.1-2019. IEEE Standard for Safety Levels With Respect to Human Exposure to Electric, Magnetic, and Electromagnetic Fields, 0Hz to 300 GHz. New York, NY: IEEE (2019).

[4] ICNIRP. Guidelines for limiting exposure to electromagnetic fields (100 kHz to 300 GHz). Health Phys. (2020) 118:483–524. doi: 10.1097/HP.0000000000001210 32167495

[5] Dagro AM Wilkerson JW Thomas TP Kalinosky BT Payne JA. Computational modeling investigation of pulsed high peak power microwaves and the potential for traumatic brain injury. Sci Adv. (2021) 7:44. doi: 10.1126/sciadv.abd8405 34714682 PMC8555891

[6] Lin JC. Auditory Effects of Microwave Radiation. Springer, Switzerland (2021) doi: 10.1007/978-3-030-64544-1

[7] Lin JC. The microwave auditory effect. IEEE J Electromagn RF Microwaves Med Biol. (2022) 6:16–28. doi: 10.1109/JERM.2021.3062826

[8] Lin JC. Microwave auditory effects among US government personnel reporting directional audible and sensory phenomena in Havana. IEEE Access. (2022) 10:44577–82. doi: 10.1109/ACCESS.2022.3168656

[9] Relman DA. Neurological illness and national security: lessons to be learned. JAMA. (2024) 331:1093–5. doi: 10.1001/jama.2023.26818 38497785

[10] Seaman RL Beblo DA Raslear TG Modification Modification of acoustic and tactile startle by single microwave pulses. Physiol Behav. (1994) 55:587–95. doi: 10.1016/0031-9384(94)90121-X 8190781

[11] Brown DO Lu ST Elson EC. Characteristics of microwave evoked body movements in mice. Bioelectromagnetics. (1994) 15:143–61. doi: 10.1002/bem.2250150206 8024606

[12] Lin JC. New ICEMAN project seeks answers to fighter pilot disorientation. IEEE Microwave Magaz. (2021) 22:13–5 doi: 10.1109/MMM.2020.3048210

[13] Cavarnaro M Lin JC. Importance of exposure duration and metrics on correlation between RF energy absorption and temperature increase in a human model. IEEE Trans Biomed Eng. (2019) 66:2253–8. doi: 10.1109/TBME.2018.2886475 30561338

[14] ICNIRP Statement - gaps in knowledge relevant to the “ICNIRP Guidelines for limiting exposure to time-varying electric magnetic and electromagnetic fields (100 Hz to 300 GHz). Health Phys. (2025) 128:190–202. doi: 10.1097/HP.0000000000001944 39670836

[15] Mevissen M Ducray A Ward JM Kopp-Schneider A McNamee JP Wood AW . Effects of radiofrequency electromagnetic field exposure on cancer in laboratory animal studies, a systematic review. Environ Int. (2025) 199:109482. doi: 10.1016/j.envint.2025.109482 40339346

[16] Melnick RL Moskowitz JM Héroux P Mallery-Blythe E McCredden JE Herbert M . on behalf of the International Commission on the Biological Effects of Electromagnetic Fields (ICBE-EMF). The WHO-commissioned systematic reviews on health effects of radiofrequency radiation provide no assurance of safety. Environ Health. (2025) 24:70. doi: 10.1186/s12940-025-01220-4 41034851 PMC12490090

